# 752 Burn Rehab Guidelines Following Application of Cultured Epithelial Autografts For Closure of Large Burn Wounds

**DOI:** 10.1093/jbcr/irad045.227

**Published:** 2023-05-15

**Authors:** Sarah Sabbatini, David Hill

**Affiliations:** Firefighters Burn Center, Memphis, Tennessee; Regional One Health, Memphis, Tennessee; Firefighters Burn Center, Memphis, Tennessee; Regional One Health, Memphis, Tennessee

## Abstract

**Introduction:**

As the use of CEA by burn surgeons becomes more widespread, the existence of an experienced and dedicated burn therapy team, educated in post-operative care and treatment of CEA, is essential to short and long-term success of patients post-CEA, and their ability to achieve and maintain maximum functional status. Early mobilization of patients is an important aspect of treatment in the BICU, and has demonstrated a positive effect on patient outcomes throughout the continuum of care. However, postoperative treatment practices following application of CEA vary across burn centers, depending on experience and perceptions of CEA fragility and expense.

The objective of this case series is to review one burn center’s therapy treatment plan before and after CEA application, and short and long-term outcomes in response to early implementation of therapy interventions. The hypothesis is early mobilization of patients after CEA can be achieved without negative consequence of graft loss.

**Methods:**

This single-center, retrospective case series utilized electronic chart review to assess patients admitted between Apr '15 and Apr '20 who received CEA. Demographics, injury characteristics, surgical procedures collected. Therapy interventions and outcomes also collected, including postoperative therapy treatment plans, functional status at and following discharge, and occurrence of graft loss and contracture formation. Data collected analyzed with descriptive statistics.

**Results:**

8 patients met inclusion criteria for the case series. Median post-operative day for initiating positioning techniques post CEA application was POD0 (0,0); POD6.5 (3.75,7) for active, passive elongation; POD12 (11,15) for out of bed mobilization, functional mobility training; POD25 (17.75,25) for return to treatment in therapy clinic. All 8 patients demonstrated 0% occurence of graft loss and 0% contracture formation; none of the patients required reconstructive surgical procedures as a result of decreased joint mobility. In addition, 6 out of the 8 patients were admitted to the burn-specific inpatient rehab program, and all achieved a functional status of independent community locomotor upon discharge, including return to work and leisure activity.

**Conclusions:**

Early implementation of therapy interventions by burn therapists trained in post-op treatment of CEA resulted in optimal short and long-term functional patient outcomes following closure with CEA. Early mobilization of patients in the BICU post-CEA did not result in any graft loss related to therapy treatment, despite the increased risk for shearing due to the physiological structure of CEA. 75% of patients reviewed in this case series achieved functional mobility skills required for independent living upon discharge, as well as return to work or leisure activities, after receiving treatment in a burn-specific inpatient rehabilitation facility within the burn center.

**Applicability of Research to Practice:**

n/a

